# Supplementary material for: Amphipathic Solvent-Assisted Synthetic Strategy for Random Lamellae of the Clinoptilolites with Flower-like Morphology and Thinner Nanosheet for Adsorption and Separation of CO2 and CH4
Source: Nanomaterials (Basel). 2023 Jun 26;13(13):1942. doi: 10.3390/nano13131942 (PMC10343432; doi:10.3390/nano13131942)
Supplement: Supplementary file 1 [file nanomaterials-13-01942-s001.zip › nanomaterials-2146367-supplementary.pdf]

**Amphipathic solvent-assisted synthetic strategy for random lamellae of the clinoptilolites with flower-like morphology and thinner nanosheet for adsorption and separation of $CO_2$ and $CH_4$**

Jiawei Zhou, Bingying Jia, Bang Xu*, Jihong Sun*, Shiyang Bai

Beijing Key Laboratory for Green Catalysis and Separation, Department of Chemical Engineering, Beijing University of Technology, Beijing, 100124, China

**Electronic Supporting Information**

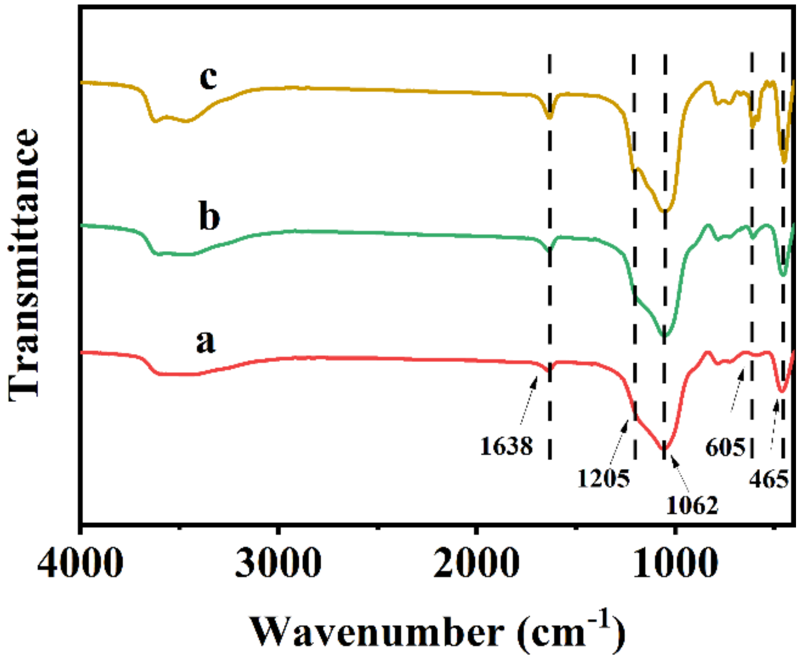


**Figure S1.** FT-IR spectra of CP-0.03 synthesized CPs with different crystallization time: 12 h (a), 36 h (b) and 72 h (c).

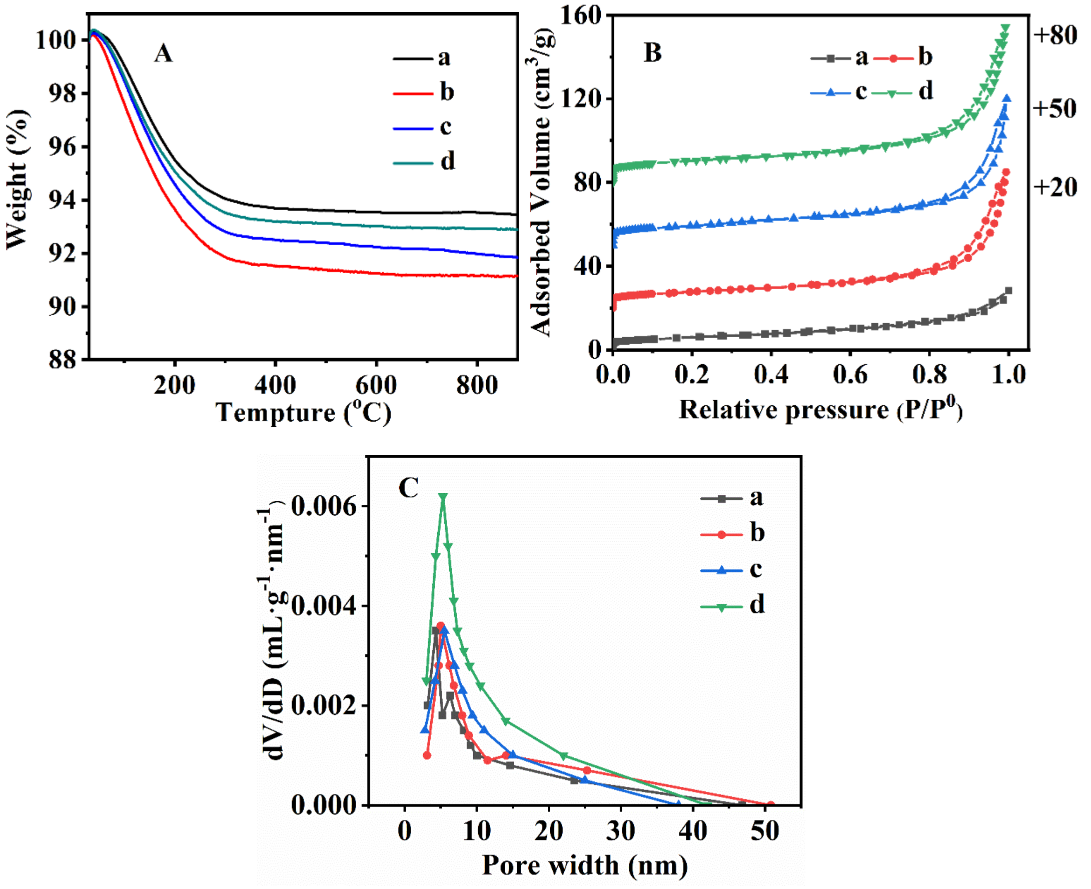


**Figure S2.** TG curves (A), $N_2$ adsorption-desorption isotherms (B) and Pore size distribution (C) of (a) parent CP, (b) CP-0.03, (c) CP-0.03-U and (d) CP-0.03-U-E.

**Table S1.** Summary of textural properties for various samples.

| Sample | BET surface area ($m^2 \cdot g^{-1}$) | Micropore surface area ($m^2 \cdot g^{-1}$) | External surface area ($m^2 \cdot g^{-1}$) | Pore Volume ($mL \cdot g^{-1}$) |
|---|---|---|---|---|
| CP | 20.4 | 3.2 | 17.2 | 0.044 |
| CP-0.03 | 27.1 | 7.8 | 19.3 | 0.101 |
| CP-0.03-U | 32.8 | 5.6 | 27.2 | 0.108 |
| CP-0.03-U-E | 36.7 | 15.9 | 20.8 | 0.115 |
